# Sending science down yonder

**DOI:** 10.1172/JCI196866

**Published:** 2025-07-15

**Authors:** Anna Bright

**Affiliations:** Icahn School of Medicine at Mount Sinai, New York, New York, USA.

“You’re from here and you’re doing that?” I turn to face the 13-year-old questioning me. I’m standing amidst thirty eighth graders in my rural hometown’s public middle school, a beige concrete block of a building whose hallways shrink with each visit. The disbelief in his voice strikes me most — the doubt that someone from a town where the cows outnumber the people could be pursuing a doctoral degree in neuroscience. “Yes,” I reply, “and I’m not anything special.”

Being born to a blue-collar family in rural Tennessee afforded me several learning opportunities — cattle maintenance, the basic mechanics of a mid-60s Ford Mustang, and metal welding, to name a few. Communities like mine flourish with educational resources for the manufacturing and agricultural trades, career paths that have been foundational to their social and economic success. I was surrounded by people prepared to guide me into a life of crop rotation or carburetor repair, but was left wanting for someone to explain where I could apply my propensity for biology. I was woefully underprepared for the educational and mental challenges my undergraduate neuroscience degree presented. If my degree had not required a semester of research, I would have never willingly entered a laboratory and fallen in love with the scientific process that continues to propel my career. Put simply, I did not know my options. Whether it was fate’s intervention or mere coincidence, I cannot help but feel that I stumbled upon rather than chose the profession that my passions and propensities demanded.

My story is not unique. Countless rural students face similar struggles, navigating their education with limited exposure to careers beyond their immediate surroundings. The question is, how many potential scientists are lost because they have never seen a path forward? What if, instead of stumbling upon a passion by chance, students had direct access to mentors who could illuminate the possibilities before them? This is where a structured mentorship program, built on the power of community and shared experience, can change the trajectory of young minds in rural areas.

I propose implementing mentorship initiatives in rural school systems that connect students with alumni who have pursued advanced degrees in science and medicine. In this way, they can step into shoes that have already walked their path. Existing programs disproportionately serve more urban and resourceful schools, reinforcing a cycle that limits scientific accessibility in hometowns like mine. This program’s innovation lies within its relatability — pairing current students with mentors who share the same roots will foster deeper connections and a more tangible sense of possibility.

This program’s success hinges upon a strong collaboration with local science teachers. I can personally attest to how enthusiastic these instructors are to welcome alumni who have pursued careers in science and medicine back into their classrooms. I have been invited to share my story with multiple classes at the middle and high school level. As the daughter of two high school teachers, I know that these moments are invaluable to educators, reinforcing their dedication to sparking curiosity and ambition in their classrooms. And for me, nothing has been more rewarding than seeing students’ eyes alight with possibility in my hometown. Watching students identify with my story, ask eager questions, and imagine themselves in a lab coat or research setting reminds me why representation matters.

This initiative will begin with outreach to recruit alumni and teachers. By partnering with local schools’ science teachers, mentors will be able to customize a curriculum that is tailored to the age and intellectual stage of each classroom. Whether meetings are held in person or virtually, they can be a hybrid of career exploration and personal storytelling, hands-on science engagement, and college and career readiness. By keeping sessions engaging and relatable, this program will bridge the ever-widening gap between rural students and professional scientific networks. And perhaps most importantly, it would empower students to explore how they can transition their passions into a lifetime of personal and intellectual fulfillment. In an age where students are more likely to be immersed in the latest TikTok trend than in a textbook, it is essential to show them that education is not just an obligation — it’s a privilege and a gateway to opportunity.

Science flourishes when watered by diverse perspectives. This initiative will empower students from rural backgrounds and diversify the scientific community. Scientists from rural areas often have firsthand knowledge of environmental and health care challenges outside of urban centers, meaning that their perspectives can lead to novel solutions. Science and medicine must reflect and serve all populations — I envision a future where a diverse, empowered generation of scientists make research more inclusive, equitable, and relevant to all, ultimately improving outcomes and advancing society as a whole.

## Version 1 07/15/2025

Electronic publication

